# Molecular Mechanisms of Somatic Cell Cloning and Other Assisted Reproductive Technologies in Mammals: Which Determinants Have Been Unraveled Thus Far?—Current Status, Further Progress and Future Challenges

**DOI:** 10.3390/ijms252413675

**Published:** 2024-12-21

**Authors:** Marcin Samiec

**Affiliations:** Department of Reproductive Biotechnology and Cryoconservation, National Research Institute of Animal Production, Krakowska 1 Street, 32-083 Balice, Poland; marcin.samiec@iz.edu.pl

Taking into consideration recent reports on the successful creation of cloned rhesus monkeys [1,2,3,4], a total of 25 mammalian species representing not only livestock, laboratory, companion and other domesticated animals, but also free-living animals, have been generated by somatic cell nuclear transfer (SCNT)-based cloning [5,6,7,8,9,10,11,12,13,14,15,16,17]. In spite of this fact, the effectiveness of SCNT-mediated assisted reproductive technologies (ARTs) remains at a disappointingly low range, oscillating between 0.1% and a maximum level of 5% as estimated by the rate of the propagated progeny to the number of enucleated oocytes (ooplasts) reconstructed with somatic or stem cell nuclei [3,16,17]. In this context, remarkably higher developmental outcomes of mammalian embryos and conceptuses are noticed for other ARTs focused on the extracorporeal production of embryos by either the non-assisted in vitro fertilization (IVF) of oocytes with capacitated and hyperactivated single spermatozoa or IVF assisted by intraooplasmic sperm microinjection [18,19]. Nevertheless, the developmental competences of cloned and in vitro-fertilized embryos and conceptuses seem to be profoundly mitigated in relation to the ARTs focused on the intracorporeal production of embryos [20,21]. As a consequence, it is worth highlighting that a multifaceted characterization of the parameters exerting considerable impacts on the developmental outcomes and functional attributes of SCNT- and IVF-derived embryos is indispensable to comprehensively unravel a wide variety of biological, biotechnological, molecular, transcriptomic, proteomic and epigenetic factors determining the efficiency of SCNT-based cloning and other strategies of in vitro embryo production (IVP) [17,22,23,24]. 

The complex IVP procedures in mammalian species comprise multiple steps, such as:

(1) In vitro meiotic maturation (IVM) of prophase I dictyotene-stage oocytes [25,26,27].
(2) Propagation of diploid zygotes from IVM-derived nuclear recipient oocytes achieved by either (A) standard IVF of meiotically matured oocytes via their co-incubation with motile sperm cells [28,29,30,31] or (B) their microsurgically assisted IVF accomplished by intracytoplasmic sperm injection (ICSI) followed by ectopic activation of ICSI-fertilized oocytes [32,33,34] or (C) reconstruction of enucleated metaphase II (MII)-stage oocytes (ooplasts) as a result of SCNT-based cloning completed by direct intraooplasmic injection of nuclear donor somatic/stem cells or their karyoplasts, or alternatively completed by the electrofusion of ooplasts with subzonally injected nuclear donor cells followed by the artificial activation of an embryo-specific developmental program of SCNT-generated oocytes [11,14,35,36].
(3) In vitro culture (IVC) of cloned or IVF/ICSI-derived embryos up to the morula/blastocyst stages prior to the possibility of their surgical/non-surgical transfer into reproductive tracts of surrogate females [37,38,39,40,41].

Considering the multi-complexity of IVP-mediated ARTs, a broad spectrum of determinants exert impact on the efficiency of propagating nuclear-transferred or ex vivo-fertilized embryos [17,20,23]. A predominant role is played by the parameters of functional quality pinpointed for nuclear recipient oocytes at the MII stage, which are undoubtedly correlated with a synergistic crosstalk of molecular factors responsible for the meiotic, cytoplasmic and epigenomic maturation of female gametes under in vitro culture conditions [42,43,44,45,46]. It is noteworthy that a pivotal influence is exerted by the approaches to extrinsically stimulate the embryonic developmental program of SCNT- or ICSI-reconstructed oocytes by using artificial (physical, chemical, physico-chemical or biological) activators [33,47,48,49,50,51]. Not without significance, also, is the molecular susceptibility of donor nuclear genomes inherited from somatic/stem cells or spermatozoa to functionally remodel and reprogram their transcriptomic and epigenomic signatures in host ooplasm, and subsequently in corresponding cytoplasm within blastomeres of cloned or in vitro-fertilized embryos [52,53,54,55]. In turn, the competences of donor cell nuclei to be epigenetically reprogrammed and transcriptionally activated in SCNT- or IVF/ICSI-generated embryos are largely dependent on the incidence of efficient maternal-to-embryonic transition for controlling gene expression, which is inextricably linked to the capabilities of donor nuclear genomes to successfully overcome the developmental hindrance related to cleavage blocking of extracorporeal embryogenesis [56,57,58]. Moreover, taking nuclear donor somatic or stem cells into account, and the extent of transcriptional reprogramming within their gene expression profiles during the ex vivo development of cloned embryos, the wide array of parameters is of great importance, putting the highest emphasis on

(1) The anatomo-histological origin of fetal or adult cells serving as a source of genomic DNA for the reconstruction of enucleated oocytes [59,60,61,62,63,64];
(2) The degree of cytodifferentiation or the extent of nondifferentiation/stemness determining the inheritance of epigenetic memory encoded in donor nuclei after their transplantation into host ooplasm [65,66,67,68,69];
(3) The abilities of somatic/stem cell-descended nuclear genomes to ontogenetically dedifferentiate or rearrange their transcriptomic landscapes due to the molecular erasure of donor cell-specific epigenomic obstacles/barriers within reprogramming-resistant regions enriched for transcriptionally repressive epigenetic marks, which have been found to exhibit resilience to the onset of embryonic genome activity [70,71,72,73,74,75].

Furthermore, the inter-genomic, inter-transcriptomic and inter-proteomic communication between nuclear and mitochondrial (mt) compartments and the degree of mtDNA heteroplasmy arising from the hybridization of nuclear donor cytoplasm-inherited and nuclear recipient ooplasm-descended mitochondrial genotypes (mitotypes) represent another set of molecular determinants that remarkably affect the ex vivo developmental potential and quality attributes of IVP-derived embryos created by SCNT or IVF/ICSI [76,77,78,79,80,81,82,83,84].

To summarize, this Special Issue provides not only insightful interpretation, but also topical and coming trends targeted at meticulously unveiling and explicating the role of intracellular hallmarks and the molecular interplay of cytophysiological, transcriptomic, proteomic and epigenomic factors reciprocally determining the efficiency of propagating and multiplying in vitro-generated embryos, conceptuses and progeny with the aid of SCNT-mediated cloning or IVF/ICSI-based IVP procedures, including comparative and mechanistic insights underlying in vivo embryo production. Deciphering the above-mentioned molecular networks resulting from inter-transcriptomic, inter-proteomic and inter-epigenomic crosstalk between factors affecting somatic cell cloning and a variety of other ARTs has been found to be a sine qua non condition for an increase in developmental outcomes and quality parameters of nuclear-transferred, IVF/ICSI-derived and in vivo-produced embryos. As a consequence, this contributes to a critical turning point inevitable for expedited and intensified, but also multi-faceted, inter-disciplinary and translational implementation of different IVP-based ARTs and their counterparts entailing in vivo embryo production to an extensive range of biomedical, biopharmacological, oncological, pathophysiological, biotechnological, embryological and agricultural research fields, as well as to eco-zoological studies aimed at the assessment and ex situ protection of biological diversity and, finally, to embryonic/adult stem cell research focused on the preclinical or clinical establishment of pluri-directionally differentiated cell derivatives in the most advanced, i.e., homeothermic vertebrates (mammals and aves) [16,17,23,54,75,85,86,87,88].
